# The Influence of Pedo-Climatic Conditions on the Micromorphological, Phytochemical Features, and Biological Properties of Leaves of *Saponaria sicula* Raf

**DOI:** 10.3390/ijms241411693

**Published:** 2023-07-20

**Authors:** Laura Cornara, Paola Malaspina, Federica Betuzzi, Emilio Di Gristina, Manuela D’Arrigo, Mariarosaria Ingegneri, Domenico Trombetta, Antonella Smeriglio

**Affiliations:** 1 Department of Earth, Environment and Life Sciences (DISTAV), University of Genova, 16132 Genova, Italy; laura.cornara@unige.it (L.C.); federica_betuzzi@libero.it (F.B.); 2 Department of Agricultural, Food and Forest Sciences (SAAF), University of Palermo, 90128 Palermo, Italy; emilio.digristina@unipa.it; 3 Department of Chemical, Biological, Pharmaceutical and Environmental Sciences (ChiBioFarAm), University of Messina, 98166 Messina, Italy; manuela.darrigo@unime.it (M.D.); mariarosaria.ingegneri@unime.it (M.I.); domenico.trombetta@unime.it (D.T.)

**Keywords:** *Saponaria sicula* Raf., soapwort, pedo-climatic conditions, anatomical features, micromorphology, secondary metabolites, phytochemical profile, polyphenols, antioxidant activity, anti-inflammatory activity

## Abstract

*Saponaria sicula* Raf. grows in Sicily, Sardinia, and Algeria on limestone cliffs and volcanic sands 1300–2500 m above sea level. The aim of the present study was to investigate how the pedo-climatic conditions influence the micromorphological, phytochemical, and biological properties of Sicilian *S. sicula* leaves collected in the Madonie Mountains (SsM) and on Etna Mt (SsE). Micromorphological investigations revealed that leaves from SsM had a higher amount of calcium oxalate druses in the mesophyll and a more intense blue–green staining with Toluidine blue O, indicating a higher content of polyphenols. These data were confirmed by phytochemical analyses carried out on hydroalcoholic extracts, which showed a higher content of total phenols (8.56 ± 0.57 g GAE/100 g DE) and flavonoids (6.09 ± 0.17 g RE/100 g DE) in SsM. Sixty-four compounds were identified by LC-DAD-ESI-MS analysis with propelargonidin dimer as the most abundant compound (10.49% and 10.19% in SsM and SsE, respectively). The higher polyphenol content of SsM leaves matches also with their biological activity, identifying SsM extract as the strongest plant complex (IC_50_ 2.75–477.30 µg/mL). In conclusion, the present study experimentally demonstrates that not only climatic differences but also soil characteristics affect the micromorphological, phytochemical, and biological features of this plant species.

## 1. Introduction

The genus *Saponaria* L. belongs to the Caryophyllaceae family and comprises 42 accepted species [1] that are distributed in temperate Eurasia, chiefly in the Mediterranean and Irano-Turanean region [2]. Saponaria species are perennial wild plants, commonly known as soapworts [3], and they are cultivated in various parts of the world as ornamental plants because of their beautiful white or pink flowers [1]. The generic name Saponaria was coined by Linnaeus from the Greek word ‘sapon’ meaning ‘soap’, in relation to the use of roots or rhizomes and the leaves of some species as a substitute for soap since ancient times [1]. Indeed, Saponaria species contain triterpenoid natural products, including saponins [4], that are also known for their anticancer, antimicrobial, insecticidal, and antioxidant properties [1]. The best-known species of the genus, *S. officinalis* L., has been used for medicinal purposes since the time of Dioscorides, mainly as an expectorant [5], cholagogue, depurative, diaphoretic, diuretic, purgative and tonic, and for the treatment of itchy skin [6].

The presence of *Saponaria sicula* Raf. (Sicilian soapwort) was reported by Chater [7] and Pignatti et al. [8] on the two largest islands of the Mediterranean Sea, namely Sicily and Sardinia, and in Algeria. *S. sicula* is a cushion-forming hemispherical caespitose hemicryptophyte, 10–15 cm tall, with procumbent to erect stems. The leaves (2.0–3.5 × 0.4–0.7 cm) are glaucous, narrowly spathulate to linear-oblanceolate, and the corymbose inflorescences bear reddish-pink flowers which are usually distributed around the cushion periphery, thus forming a wreath [7,8]. Two weakly differentiated subspecies are distributed in the southern part of the Balkan Peninsula: *S. sicula* subsp. *intermedia* (Simmler) Chater and *S. sicula* subsp. *stranjensis* (Jordanov) Chater [7,8]. In Sicily, *S. sicula* occurs exclusively in the Madonie Mountains (north-central Sicily) and on Etna Mt (east Sicily) [8]. The population of the Madonie Mountains (SsM) grows on stony carbonate slopes between 1300 and 1600 m a.s.l., while the Etna population (SsE) is present on volcanic sands and stony slopes between 1700 and 2500 m a.s.l. [9]. Recently, the possibility of micropropagation by the nodal culture of this rare species has been reported [10], though it has never been investigated from a phytochemical point of view, nor have the biological properties of its extract been investigated.

Environmental factors, such as soil and climatic conditions, can influence the growth and distribution of plant species. These factors have varying effects on the plant’s physiological and morphological characteristics. In particular, the pedo-climatic conditions influence the uptake of water and nutrients, also affecting the metabolism [11] and productivity of the plant species [12,13]. The capability of plants to counteract the negative effects of abiotic stress is often linked to secondary metabolites production. In particular, phenolic acids and flavonoids play an important role as antioxidants and can help plants to improve their tolerance to stressful conditions [14]. Therefore, the aim of the present study was to compare the anatomical and phytochemical features of the leaves from the two different populations (SsM and SsE) to highlight the influence of very different environmental and pedo-climatic conditions on plant secondary metabolites expression. In addition, the biological activities of the two hydroalcoholic extracts were tested in relation to their antioxidant and anti-inflammatory activities.

## 2. Results

### 2.1. Soil Features

**Figure 1:**
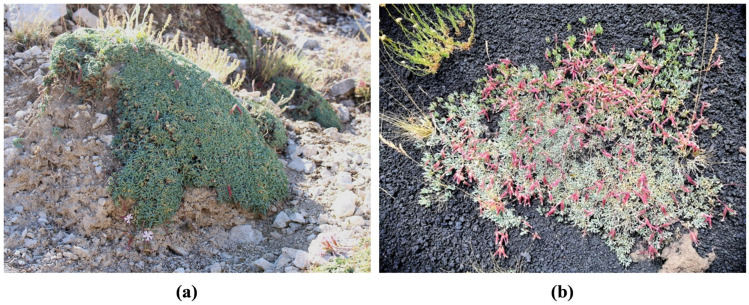
Typical cushion-forming plants of *S. sicula* growing on the stony carbonate slopes of the Madonie Mountains (SsM) (**a**) and on the volcanic sands and stony slopes of Etna Mt (SsE) (**b**).

Soils from the two collection sites were both sandy and alkaline but differed in colour, light brown for Madonie (Figure 1a) and black for Etna (Figure 1b), and composition (Table 1). The main differences were the amounts of CaCO_3_, Ca, and Mg which were very high in the Madonie soil (916.8 g/kg, 860 mg/kg, and 135.6 mg/kg, respectively), and low in the Etna soil (4.5 g/kg, 50 mg/kg, and 8.4 mg/kg, respectively). In addition, organic matter and C/N were higher in Madonie (2.4 g/100 g and 13.6, respectively) than in Etna (0.9 g/100 g and 7.1, respectively) soil.

### 2.2. Micromorphological Characterisation

**Figure 2:**
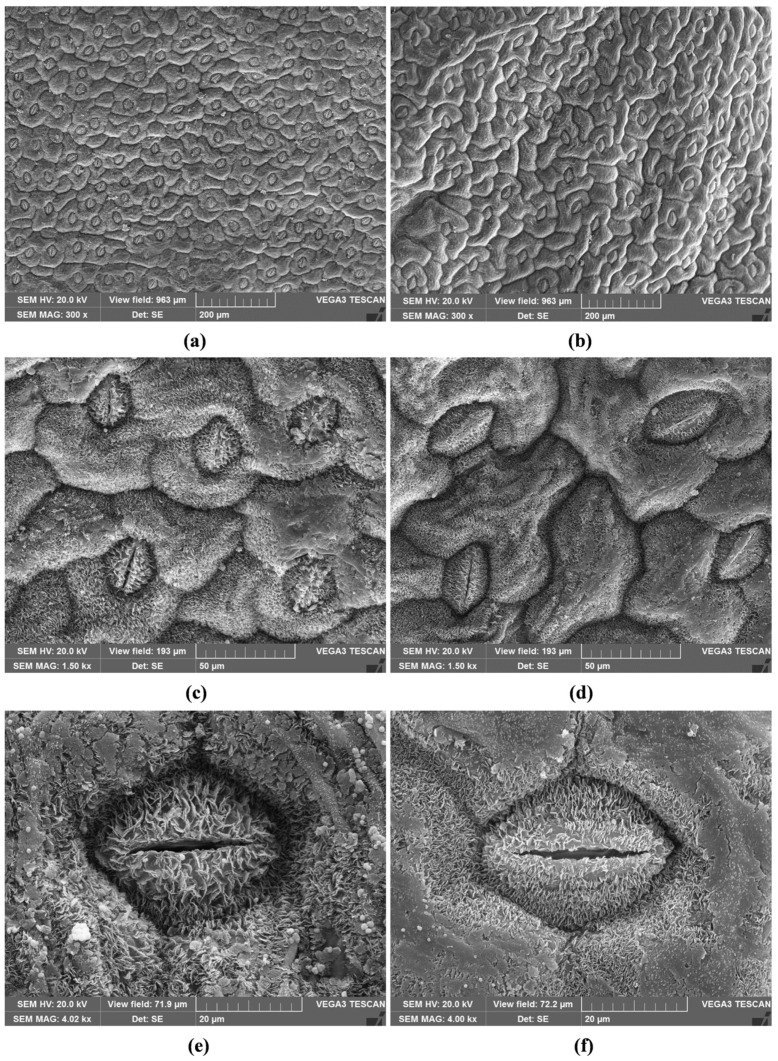
Scanning electron microscopy (SEM) micrographs of the leaf epidermis of *S. sicula* collected on Madonie (**a**–**f**). Adaxial surface (**a**,**c**,**e**) and abaxial surface (**b**,**d**,**f**). Waxes in the form of small platelets are well visible on the stomata and epidermal cells at higher magnifications (**e**,**f**).

**Figure 3:**
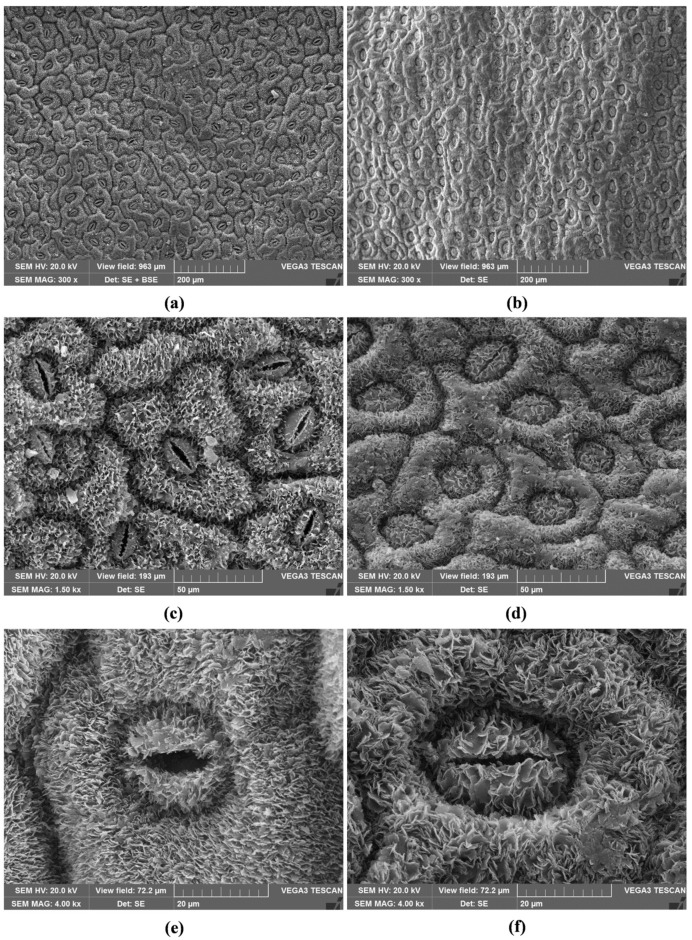
SEM micrographs of the leaf epidermis of *S. sicula* collected on Etna (**a**–**f**). Adaxial surface (**a**,**c**,**e**) and abaxial surface (**b**,**d**,**f**). Waxes in the form of small platelets are well visible on stomata and epidermal cells at higher magnifications (**e**,**f**).

The leaves of the two populations differed in size, with SsM measuring 2.0 ± 0.19 × 0.6 ± 0.08 cm, and SsE measuring 1.5 ± 0.15 × 0.4 ± 0.1 cm. *S. sicula* leaves were amphistomatic, with diacytic stomata (also known as caryophyllaceous stomata) on both epidermal surfaces, with 2–3 subsidiary cells (Figure 2a–d, SsM; Figure 3a–d, SsE).

**Figure 4:**
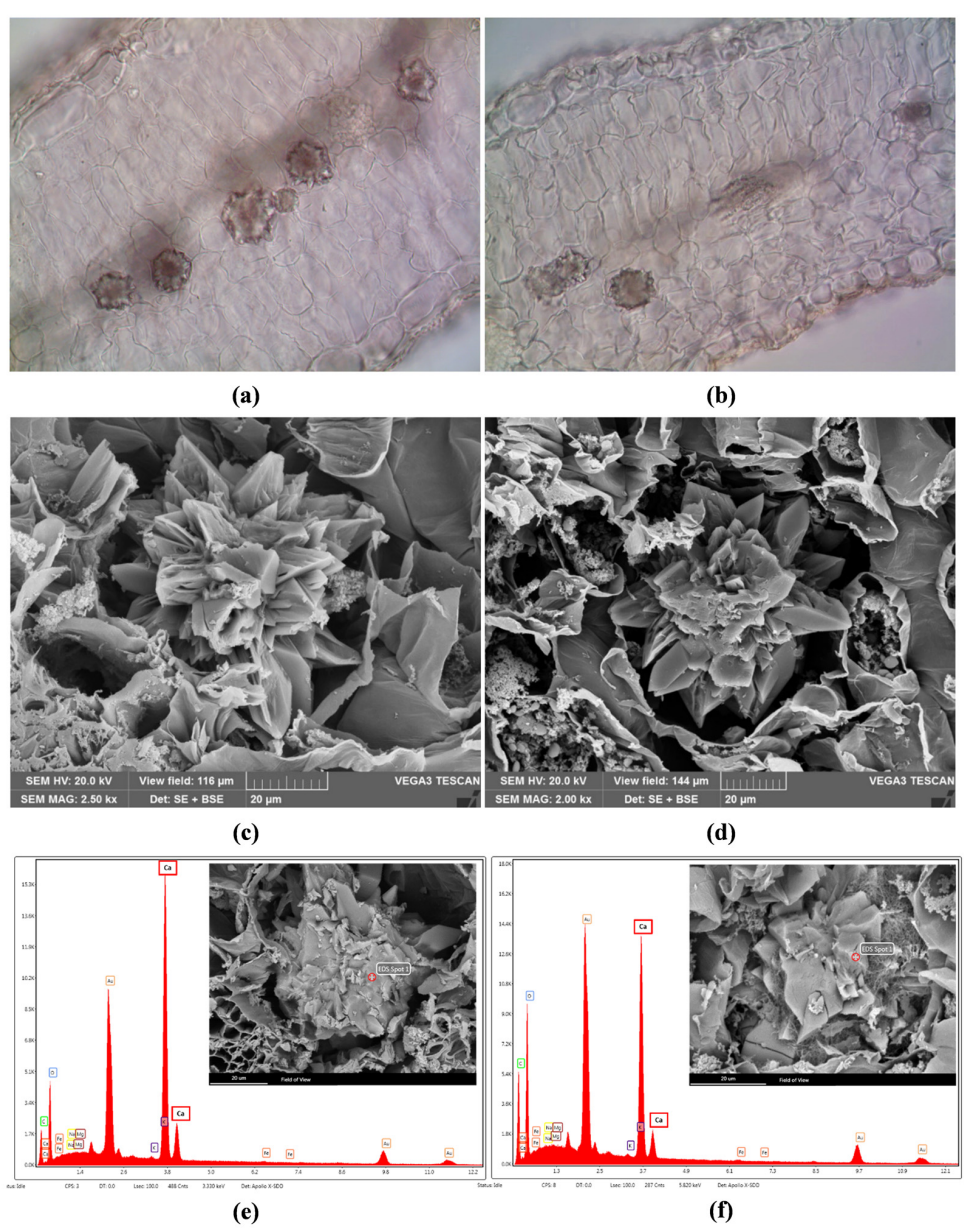
Light microscopy (LM) (**a**,**b**) and SEM (**c**,**d**) micrographs of the leaf transversal section of *S. sicula* collected on Madonie (**a**–**c**) and Etna (**b**,**d**). Calcium oxalate druses in the central portion of the mesophyll (**a**,**b**), and druses at higher magnification are shown by SEM (**c**,**d**). SEM–EDS structural characterisation of a calcium oxalate druse within the leaf mesophyll in *S. sicula* collected on Madonie (**e**) and Etna (**f**). The “Au” peak corresponds to the gold coating of the sample. The “Ca” peak indication is highlighted. Bars in (**a**,**b**): 100 µm.

The stomata were slightly sunken, showing xeromorph features (Figure 2c–f, SsM; Figure 3c–f, SsE). Waxes, which cover epidermal leaf surfaces, consisted of platelets that were usually broader than high, appearing less densely clustered and prominent in SsM than in SsE (Figure 2e,f, SsM; Figure 3e,f, SsE). In the transversal section, the leaves from both populations showed an isobilateral mesophyll with highly packed cells, characterised by small intercellular spaces, so that the palisade and spongy parenchyma were not clearly distinguishable (Figure 4a,b).

**Figure 5:**
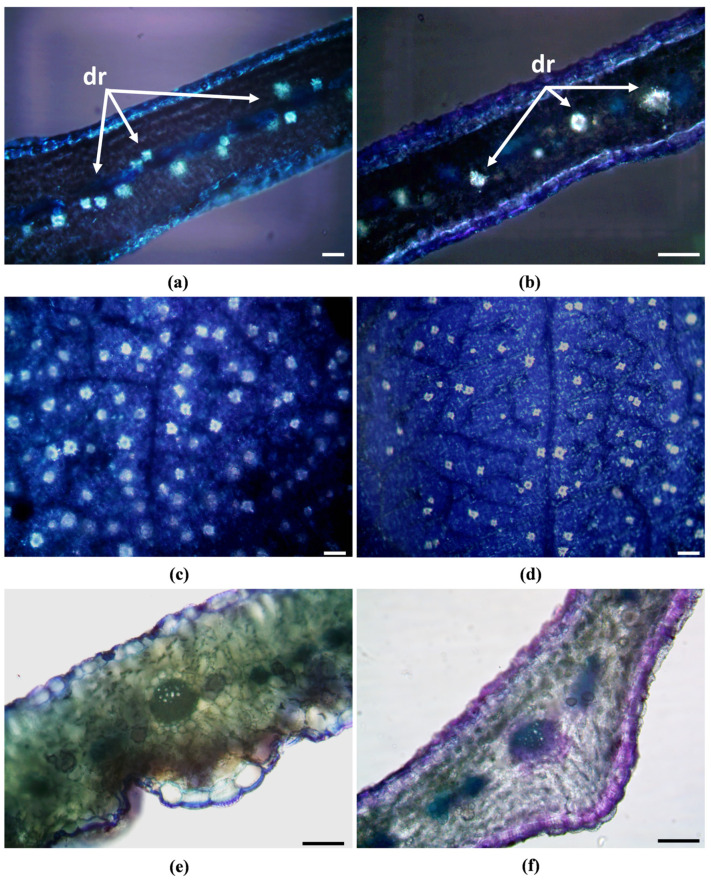
LM images under polarised light. Hand-made leaf transversal sections (**a**,**b**); clarified leaves (**c**,**d**). Hand-made transversal sections stained with TBO (**e**,**f**): a more intense greenish-blue staining is visible in SsM (**e**) in comparison with SsE (**f**). Bars: 100 µm.

In the central portion of the mesophyll of both populations, many druses (Figure 4a–d; Figure 5a,b) were detected by both light and scanning electron microscopy (LM and SEM, respectively). Druses were well visible in polarised light in clarified leaves (Figure 5c,d), with a significantly higher number in SsM (113.27 ± 11.4) with respect to SsE (61.6 ± 8.3) (*p* < 0.0001). However, despite the different densities, the overall druse structure did not appear to be affected by the different environmental conditions. SEM coupled with energy dispersive spectroscopy (SEM–EDS) allowed for classifying the crystals as calcium oxalate druse, as shown by the largest peak of calcium, while only traces of other elements, such as magnesium and potassium, were found (Figure 4e,f). Moreover, the histochemistry analysis carried out after Toluidine Blue O (TBO) metachromatic staining revealed a higher abundance of polyphenols within the mesophyll cells of SsM in comparison with SsE, as shown by the more intense greenish-blue staining (Figure 5e,f).

### 2.3. Stomatal Index and Density

As shown in Table 2, the intra- and inter-specific difference in the stomatal index was not statistically significant. On the contrary, significant differences in stomal density (SD) were found between SsM and SsE, with SsE showing a higher mean density in both epidermal surfaces. In addition, in SsM, a significant difference in the SD between the leaf adaxial and abaxial surfaces was also observed.

### 2.4. Phytochemical Analyses

The highest polyphenol content detected in SsM by light microscopy (LM) after TBO staining was confirmed by in vitro phytochemical screening (Table 3). Indeed, the SsM hydroalcoholic extract showed the highest content of total phenols and flavonoids (*p* < 0.05, Table 3). The vanillin index, whose value decreases with the increase in polymerisation degree because many of the C-6 and C-8 positions are involved, allowed also for the quantification of the flavan-3-ols content of the two extracts, showing the highest content of this class of flavonoids in SsM (*p* < 0.05, Table 3). On the contrary, no statistically significant difference was observed between SsM and SsE for proanthocyanin content. Nevertheless, by calculating the polymerisation index of the two extracts, dividing the vanillin index by the proanthocyanidin content, two very distant values were obtained, indicating a clear difference between the two extracts in terms of monomeric molecule content, which was much more abundant in SsM (Table 3).

These preliminary data were corroborated by an in-depth phytochemical analysis carried out by LC-DAD-ESI-MS. A total of 63 compounds (54 in SsM and 57 in SsE) were detected and tentatively identified by comparison of mass and UV–Vis spectra with the literature and online free consulting spectra databases, as well as with commercially available standards or, if not available, with the most structurally similar compounds (see Table 4 footnotes for details). Considering the sum of the peaks’ areas, the SsM extract appears, once again, to be the richest in secondary metabolites. Considering the relative abundance of the identified compounds, by expressing the results as an area percentage with respect to the total area of detected peaks, the most abundant classes of identified compounds are, in order of abundance for SsM and SsE, respectively: flavonoids (54.84% and 56.43%, respectively), phenolic acids (15.45 and 16.10%, respectively), tannins (11.72% and 11.27%, respectively), and lignans (10.28% and 9.63%, respectively). Considering this, the investigated extracts showed a very similar qualitative profile, as expected from plants belonging to the same species. The main statistically significant differences (*p* < 0.05) reside in the less expressed compounds. Specifically, a higher abundance of saponins was detected in SsE (4.40% vs. 2.61% in SsM), whereas a higher expression of other compounds such as stilbenes and secoiridoids was detected in SsM (5.10% vs. 2.18% in SsE).

The proanthocyanidin propelargonidin dimer (10.49% and 10.19% in SsM and SsE, respectively) and phenolic acid sinapoyl-feruloyl-gentiobiose (6.54% and 7.43% in SsM and SsE, respectively) are the most abundant compounds in both extracts, followed by caffeoyl glucose (5.60%), petunidin (5.52%), and lariciresinol-sesquilignan (5.47%) in SsM, and lariciresinol-sesquilignan (4.72%), caffeoyl glucose (4.33%), and kaempferol-*O*-feruloyl-sophoroside (3.83%) in SsE.

**Figure 6:**
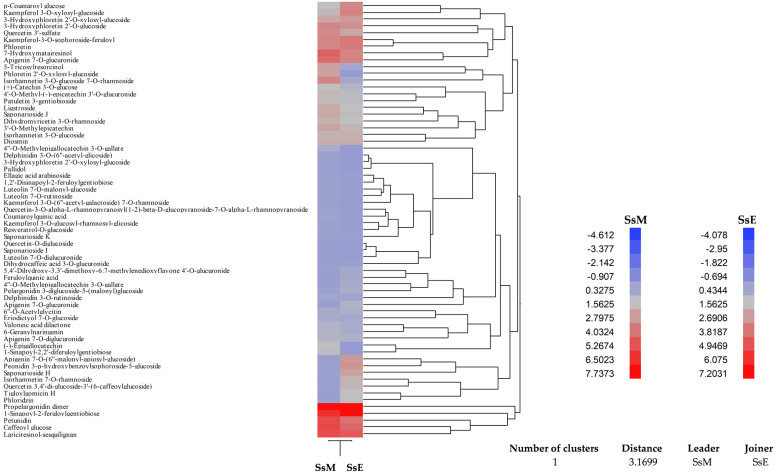
Agglomerative hierarchical clustering analysis of the phytochemical data of *S. sicula* collected on Madonie and Etna (SsM and SsE, respectively) obtained by the LC-DAD-ESI-MS analysis of the two hydroalcoholic extracts. The heatmap shows the expression pattern of the identified metabolites, indicating in red and blue the most and the least expressed metabolites, respectively. The colour density indicates the fold change between the investigated extracts.

In any case, beyond the qualitative profile, what substantially changes is the level of expression of each metabolite, as can be seen from the agglomerative hierarchical clustering analysis (Figure 6). Indeed, the resulting heatmap shows the expression pattern of the identified metabolites, indicating in red and blue the most and the least expressed metabolites, respectively. The colour density indicates the fold change between the investigated extracts, allowing us to observe easily and immediately how the expression of each metabolite of the same plant species grown in different pedo-climatic conditions changes, sometimes even conspicuously. Indeed, it is interesting to observe, as can be seen from Table 4 and even better from the heatmap shown in Figure 6, that eight metabolites (feruloylquinic acid, apigenin-*O*-glucuronide, apigenin-*O*-(malonyl-apiosyl-glucoside, quercetin-*O*-diglucoside, saponarioside I, dihydrocaffeic acid-*O*-glucuronide, tigloylgomicin H, and phloridzin) present in SsM were not detected in SsE. Similarly, the other four metabolites (delphinidin-*O*-(-acetyl-glycoside, pallidol, hydroxyphloretin-*O*-xylosyl-glucoside, and sinapoyl-diferuloylgentiobiose) present in SsE were not detected in SsM.

### 2.5. Biological Activity

**Figure 7:**
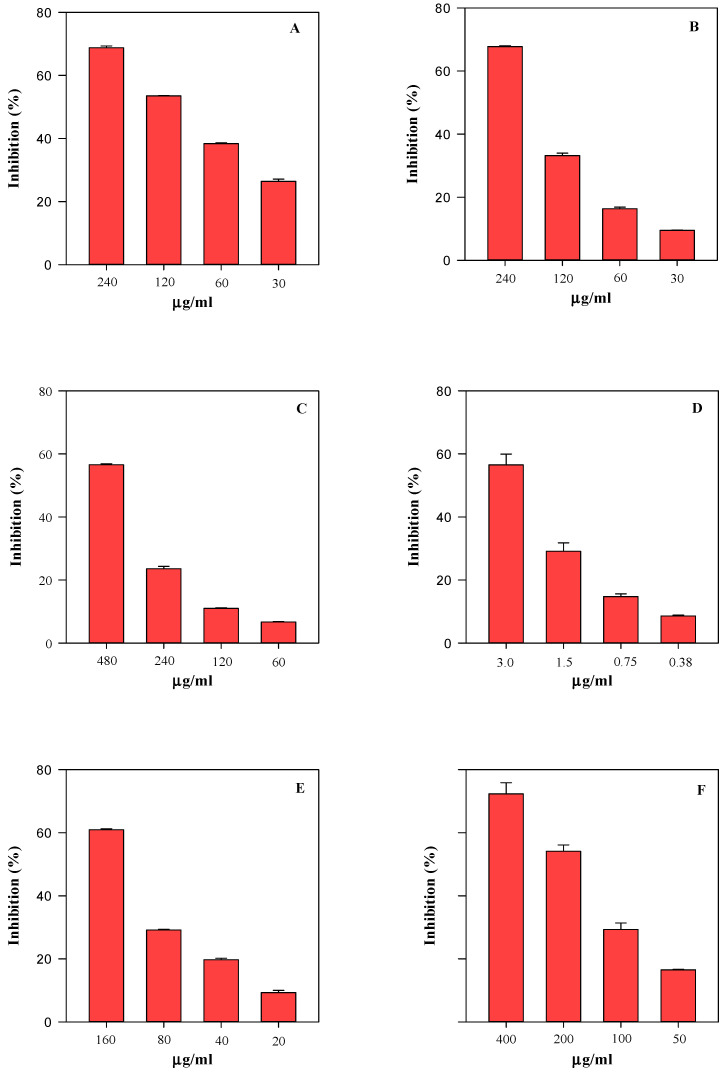
Antioxidant and free radical-scavenging concentration-dependent behaviour of *S. sicula* collected on Madonie (SsM) evaluated by TEAC (panel **A**), FRAP (panel **B**), DPPH (panel **C**), ORAC (panel **D**), ICA (panel **E**), and BCB (panel **F**) assays. The results, expressed as the inhibition (%), show the mean and standard deviation of three independent experiments in triplicate (*n* = 3).

**Figure 8:**
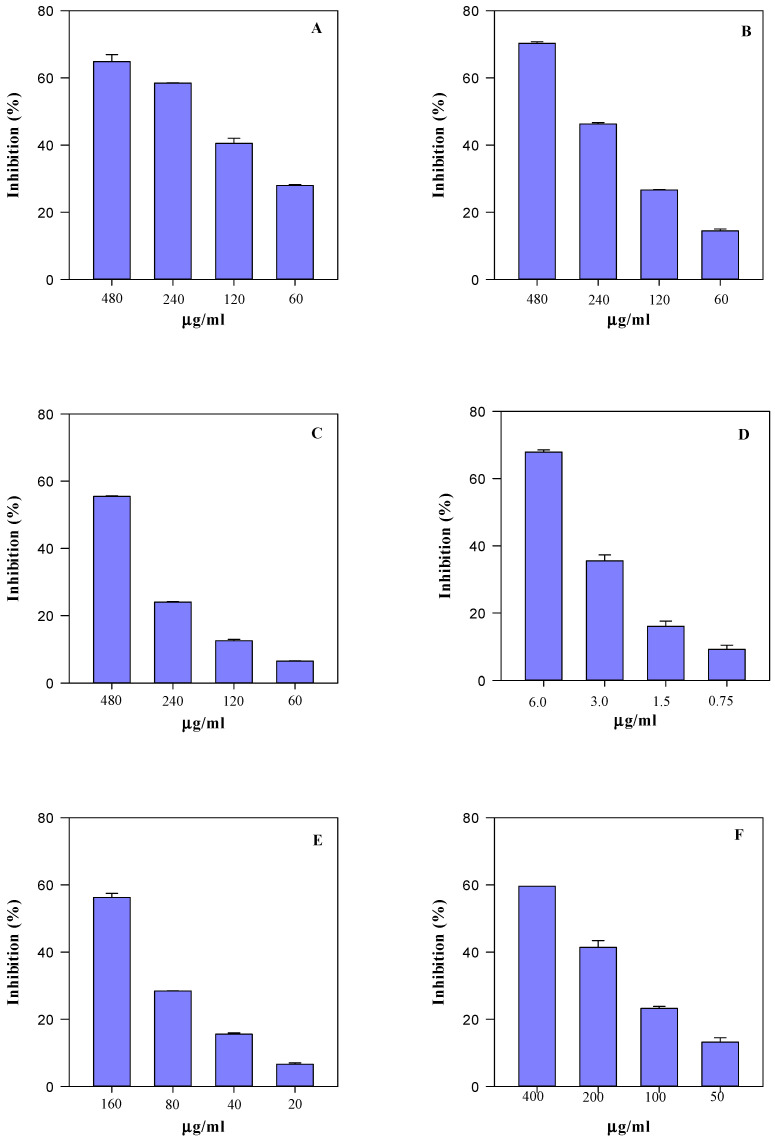
Antioxidant and free radical-scavenging concentration-dependent behaviour of *S. sicula* collected on Etna (SsE) evaluated by TEAC (panel **A**), FRAP (panel **B**), DPPH (panel **C**), ORAC (panel **D**), ICA (panel **E**), and BCB (panel **F**) assays. The results, expressed as the inhibition (%), show the mean and the standard deviation of three independent experiments in triplicate (*n* = 3).

**Figure 9:**
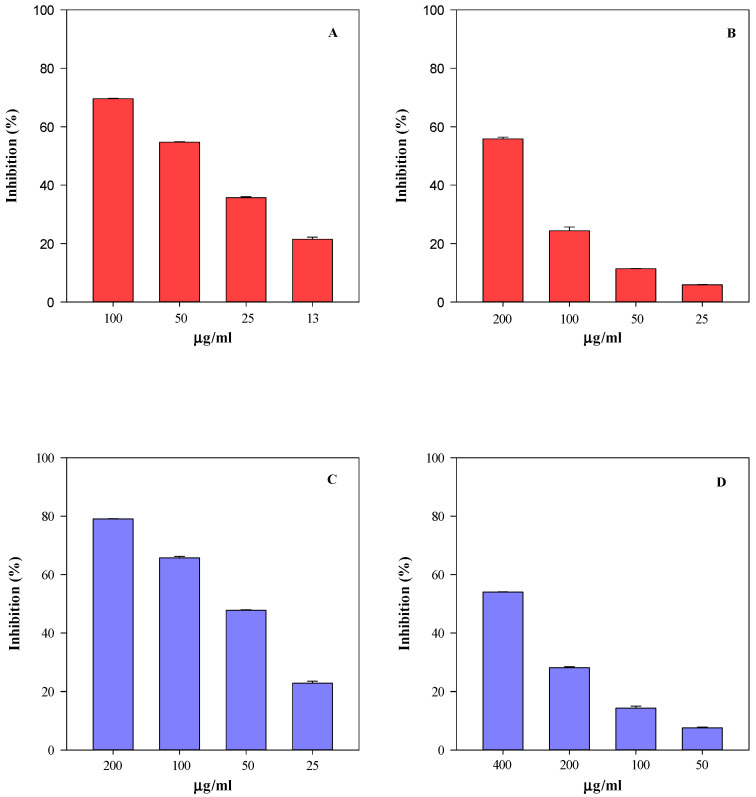
Anti-inflammatory concentration-dependent behaviour of *S. sicula* collected on Madonie (SsM) and Etna (SsE) evaluated by protease (panel **A** and **C**, respectively) and albumin denaturation (ADA) assays (panel **B** and **D**, respectively). The results, expressed as the inhibition (%), show the mean and standard deviation of three independent experiments in triplicate (*n* = 3).

In order to evaluate how the differences recorded in the phytochemical profile of SsM and SsE are reflected in their biological properties, their antioxidant and anti-inflammatory activity was evaluated by several in vitro spectrophotometric and spectrofluorimetric assays (Figure 7, Figure 8 and Figure 9).

Specifically, four tests were carried out to evaluate the sample free-radical scavenging activity, i.e., the trolox equivalent antioxidant capacity (TEAC), the ferric-reducing antioxidant power (FRAP), the scavenging activity against 2,2-diphenyl-1-picrylhydrazyl radical (DPPH), and the oxygen radical absorbance capacity (ORAC) assays. These tests aimed to evaluate the scavenging ability of the extracts against 2,2′-azino-bis(3-ethylbenzothiazoline-6-sulfonic acid), 2,4,6-tris(2-pyridyl)-s-triazine (ABTS and TPTZ, respectively), DPPH, and 2,2′-azobis(2-amidinopropane) dihydrochloride (AAPH) radicals, which are triggered by different reaction mechanisms and conditions. Furthermore, considering the conspicuous presence of monomeric compounds with free hydroxyl groups, some of which are also characterised by the catechol group (OH-groups in the ortho position), the iron-chelating activity (ICA) and the ability of samples to counteract heat-induced β-carotene bleaching (BCB) were also evaluated. Figure 7 and Figure 8 show the results of the antioxidant activity of the SsM and SsE extracts, respectively, whereas Figure 9 shows the results of the anti-inflammatory activity of the two extracts in comparison. After an initial screening in a wide concentration range, four concentrations were selected for each extract with the aim of calculating the half-maximal inhibitory concentration (IC_50_) with the respective confident limits (C.L.), as shown in Table 5. Both extracts showed a concentration-dependent behaviour (Figure 6 and Figure 7) with the following order of potency: ORAC > TEAC > ICA > FRAP > BCB > DPPH for SsM and ORAC > ICA > TEAC >FRAP > BCB > DPPH for SsE (Figure 6 and Figure 7; Table 5). The highest iron-chelating activity recorded for SsE was in accordance with the highest flavonoid content detected by LC-DAD-ESI-MS analysis.

The inflammatory process triggers various events in the human organism. Among these, the protein denaturation of biological membranes and induction of protease activity certainly play a pivotal role. Therefore, the study of the inhibitory activity of plant extracts on these two phenomena, by an albumin denaturation assay (ADA) and protease activity, may represent a simple and useful in vitro screening tool that could help to find new anti-inflammatory plant complexes.

The anti-inflammatory activity results, depicted in Figure 9, show a superimposable behaviour with respect to the antioxidant activity, with a concentration-dependent trend and SsM showing once again the strongest activity (*p* < 0.05).

The antioxidant and anti-inflammatory activity behaviour is quite similar between the two extracts. However, when analysing the IC_50_ values (Table 5), it is clear that, in accordance with the phytochemical data, the SsM extract, which is the richest in secondary metabolites, is also the strongest from both antioxidant and anti-inflammatory points of view (*p* < 0.05). Indeed, it shows the lowest IC_50_ values, thus highlighting a linear correlation between the secondary metabolite content and biological activity.

## 3. Discussion

Pedo-climatic conditions can affect plant distribution, production, and physiology, causing sometimes substantial changes from a morphological and phytochemical point of view [13].

In the leaves of both populations of *S. sicula*, a higher stomatal density on the adaxial surface was observed. This agrees with the explanation of a higher light intensity generally occurring on the leaf’s upper surface. Leaves of SsE also showed a significantly higher mean SD in both epidermal surfaces with respect to SsM. This fact is difficult to explain since a decrease in SD is considered a general response to elevated CO_2_ exposure [15], a situation commonly occurring around the Etna volcano [16]. However, some studies have highlighted that a reverse response could occur when elevated CO_2_ interacts with other climatic factors such as a higher temperature or soil properties that may limit leaf enlargement, leading to increased SD [17]. In this regard, less expanded leaves were observed on average in SsE with respect to SsM; this may explain the higher SD value observed.

Therefore, it could be conceivable that SsE, growing on volcanic soil, uses various morphological adaptations to survive in conditions that are often difficult due to the persistent and varied eruptive activity of Etna. In line with this, we observed that the waxes covering the leaf surfaces, despite having the same typical micromorphological characteristics of Caryophyllales [18], appeared more densely clustered and prominent in SsE.

From an anatomical point of view, the leaves of both populations showed a mesophyll in which palisade and spongy tissues were not well differentiated and could be described as isobilateral, according to Esau [19]. In the central portion of the mesophyll, calcium oxalate druses were found to be more numerous and closer to each other in SsM than in SsE leaves. This fact is probably related to the much higher amount of calcium and calcium carbonate detected in the Madonie soil with respect to that of Etna. The presence of calcium oxalate druses has been previously reported in other species of the *Saponaria* genus. Tütüncü Konyar et al. [20] found druses in the leaves and stem of *S. officinalis*, while Ataşlar [4] reported their presence in the leaves and roots, but not in the stem, of *Saponaria kotschyi* Boiss. The main function of these crystals is to remove excess calcium, regulating the bulk-free calcium levels in plant tissues and organs. This, according to soil features detected in this study, could explain why they were most abundant in SsM rather than in SsE. Moreover, calcium oxalate druses can also protect plants against herbivores [21].

The more intense blue-greenish colour observed in the leaves of SsM after TBO staining agreed with the higher content of polyphenols highlighted by phytochemical analyses. This is the first study that investigated the phytochemical profile and biological properties of *S. sicula*. The two sampling sites investigated in this study are very different both in terms of climate and soil characteristics and it is well known that numerous factors affect the polyphenol content of plants, including environmental factors as well as edaphic factors like soil type and sun exposure, rainfall, etc. These abiotic stresses have a negative impact on plant growth, development, and productivity [22,23,24]. Plants, being sessile, are persistently exposed to these factors and require a set of effective mechanisms to counteract these unfavourable circumstances [25]. From this point of view, the accumulation of polyphenols plays a pivotal role in plant defence [26,27], so much so that the concentration of polyphenols in plant tissue is a useful marker in predicting the extent of abiotic stress tolerance in plants belonging to the same species. Plant phenolics play an important role in several physiological processes to improve the tolerance and adaptability of plants under stress conditions [28,29,30]. They are involved, for example, in signal transduction from the root to the shoot, and help in nutrient mobilisation. The root exudates contain phenolic compounds which modify the physiochemical properties of the rhizosphere. Soil microbes transform phenolics into compounds that help in N mineralisation and humus formation [31]. Indeed, it has been demonstrated that a high polyphenol production by plants represents an adaptive response to control the nitrogen’s fate and influence the plant’s competitive advantage in the uptake of organic nitrogen. Furthermore, it has been demonstrated that polyphenols improve nutrient uptake through the chelation of metallic ions, enhancing active absorption sites and soil porosity with the accelerated mobilisation of elements like calcium (Ca), magnesium (Mg), and potassium (K) [14]. These observations are in line with the results of the present study that highlighted the highest content of polyphenols in SsM grown in soil with a higher C/N ratio, the highest values of Ca and Mg, as well as the highest cation exchange capacity.

However, beyond the role of polyphenols in terrestrial ecosystem nutrient cycling, the main role of polyphenols is that they are powerful antioxidants. Indeed, the first consequence of abiotic stresses is the production of ROSs [32,33], which are very harmful to DNA, proteins, carbohydrate, lipids, and, in general, plant cells [34]. Generally, plants counteract these events by inducing antioxidant enzyme production such as superoxide dismutase, catalase, ascorbate peroxidase, glutathione peroxidase, glutathione reductase, etc., but sometimes this is not enough to restore the physiological state, leading to an increased ROS content within the cell [35]. Following this event, polyphenols and especially flavonoids help plants to counterbalance the excessive ROS production and repair the cell damage [36,37] through the following main pathways: (i) free radical-quenching activity, (ii) down-regulation of ROS-producing enzymes such as xanthine oxidase, lipoxygenase, protein kinase C, cyclooxygenase, microsomal monooxygenase, mitochondrial succinoxidase, and NADPH oxidase [38,39], (iii) chelation of xanthine oxidase, (iv) chelation of transition metals, (v) recycling of other antioxidants, and (vi) activation of plants’ natural antioxidant enzymes [38,40,41]. Numerous abiotic stressors trigger highly hydroxylated flavonoids production. For example, quercetin-*O*- and luteolin-*O*-glycosides, have a catechol group in the B-ring of the flavonoid skeleton which shows considerable antioxidant activity in plant cells [42]. Similarly, kaempferol, a monohydroxy B-ring flavanol, also showed antioxidant properties under light irradiance [43]. However, it has been observed in most cases that quercetin derivatives are more efficient than a monohydroxy B-ring, particularly in iron-chelating activity.

It has been demonstrated that UV-B radiation increases the flavonoid content [44], that UV-B stress and drought increase the proanthocyanidin content [45], and that water stress increases the quercetin, apigenin, and luteolin-derivative contents [46]. On the contrary, heat stress seems to increase the flavanol content [47]. According to this, the higher antioxidant activity found in the SsM leaf extract can be correlated to the greater expression of these classes of polyphenols.

Generally, the difficulty in understanding how abiotic stressors affect the polyphenolic profile of plants mainly lies in the fact that some of these compounds are species-specific and their biosynthesis is dependent on the developmental stage and the nature of the stresses [48,49]. From this point of view, we believe that this type of multidisciplinary study, carried out by comparing plants belonging to the same species and grown in quite different pedo-climatic conditions, even in the same region, may help to better understand how the micromorphological, phytochemical, and biological features of a plant species changes following exposure to different abiotic stressors.

## 4. Materials and Methods

### 4.1. Chemicals

Reagents, as well as American Chemical Society (ACS) and LC-MS-grade solvents and acids, unless otherwise specified, were purchased from Merck (Darmstadt, Germany).

### 4.2. Plant Material

The fully developed basal leaves of *S. sicula* were collected, at the end of the flowering stage, directly in the field from plants growing in the Madonie Mountains and on Etna Mt in July 2022.

The plants from Madonie were collected on west-facing carbonate stony slopes at 1350 m a.s.l. (Table 6). From a bioclimatic point of view, this area falls into the Mediterranean pluviseasonal oceanic scenario, with a lower oro-mediterranean thermotype and lower humid ombrotype [50].

SsE were collected on south-facing volcanic stony slopes at 1800 m a.s.l. (Table 6). The area falls into the Mediterranean pluviseasonal oceanic bioclimate, with a lower oro-mediterranean thermotype and upper humid ombrotype [50].

For each population, 20 randomly selected individuals, separated by at least 5 m from each other, were collected. Measurements of leaf length and height were made on 10 leaves from 10 different plants for each population to compare the mean leaf size. The voucher specimens were stored in the SAF herbarium at the Department of Agricultural and Forest Science (University of Palermo) (see Table 6 for details).

To characterise the chemical and physical properties of the soils, for each collection site, soil samples (about 200 g) were taken from the rooting zone (0–20 cm). Soil samples were then air-dried at room temperature (RT) for three weeks, passed through a 2 mm sieve to remove gravel and debris, and sent to the soil testing laboratory “Regional Soil Analysis Laboratory in Sarzana” (La Spezia, Italy) (ISO 9001 certified). For each sample, routine laboratory analyses were performed in compliance with the proposed official Italian methods [51].

### 4.3. Light and Scanning Electron Microscopy

Anatomical studies were carried out on leaves preserved in FineFIX working solution (Milestone s.r.l., Bergamo, Italy) [52]. Cross-sections of the leaves were handmade by using a double-edged razor blade. Observations were made by a Leica DM 2000 transmission-light microscope (Leica Microsystems, Wetzlar, Germany), coupled with a ToupCam Digital Camera, CMOS Sensor 3.1 MP resolution (ToupTek). Polarised light was used to detect the presence and distribution of crystals within the plant tissues. For determining crystal densities, leaves were cleared with an aqueous solution of chloral hydrate and mounted in a chloral hydrate-glycerol solution to prevent crystallisation of the reagent during the observation of the slides, according to Jackson and Snowdon [53]. At least 15 random micrographs were taken at 10× magnification in the central zone of the leaf and avoiding the central midvein. For each field (1973 mm^2^), the druses density was counted using ToupView software (version x64, 4.11.20805.20220506, ToupTek Photonics, Hangzhou, China). To detect phenolic compounds, sections were treated with metachromatic staining Toluidine Blue O, pH 4.4 [54,55].

Leaves were also analysed by scanning electron microscopy (SEM) to highlight micromorphological features and to achieve a more detailed anatomical characterisation. Fixed leaves were dehydrated in a graded ethanol series (70, 80, 90, and 100%) for 1 h, and subsequently, critically point dried in CO_2_ (K850CPD 2M, Strumenti S.r.l., Roma, Italy). Small pieces and sections of the dried specimens were then mounted on aluminium stubs using two-sided adhesive carbon tape and covered with a 10-nm layer of gold particles. The specimens were examined under a VEGA3-Tescan-type LMU microscope equipped with the Energy Dispersive X-ray Spectroscopy (EDX or EDS) (Apollo, Tescan USA Inc., Cranberry Twp, PA, USA), operating at an accelerating voltage of 20 kV. EDS was used to identify the elemental composition of crystals [56].

Stomatal Index and Stomatal Density

Micrographs of SsM and SsE epidermal surfaces (adaxial and abaxial), captured by both LM and SEM, were used to determine and compare the stomatal index and stomatal density. All the photos were analysed through the free software ImageJ (v 1.53t) [57]. The stomatal index (SI) was calculated as described by Salisbury [58]:

SI = [S/(E + S)] × 100

where S = n. of stomata per unit area (mm^2^), and E = n. of epidermal cells in the same area.

Stomatal density (SD) was calculated as described by Ghosh and Davis [59]:

SD = S/A

where S = n. of stomata, and A = unit for leaf area (mm^2^).

For LM analysis, the whole leaves were bleached in a commercial 2.2% sodium hypochlorite solution (NaClO) for 60 min. Subsequently, ten random micrographs (five adaxial and five abaxial) were taken at 20× magnification, in the central zone of the leaf and avoiding the central midvein. For each field (0.468 mm^2^), stomata and epidermal cells were counted using ToupView software (version x64, 4.11.20805.20220506, ToupTek Photonics, Hangzhou, China).

For SEM analysis, four images were taken in the central zone of the leaf, avoiding the central midvein and considering both adaxial and abaxial epidermal surfaces, at different magnifications (three at 500× and one at 300×). Stomata and epidermal cells were counted in each field corresponding to 0.256 mm^2^ or 0.693 mm^2^ for 500× and 300× magnification, respectively.

### 4.4. Sample Extraction

Fully developed basal leaves of SsM and SsE were powdered by a blade mill (IKA^®^ A11, IKA^®^-Werke GmbH & Co. KG, Staufen, Germany) with liquid nitrogen to block the enzymatic activities and preserve the native phytochemical features. A food-grade extraction process was applied by adding 100 mL of an ethanol/water mixture (80:20, *v*/*v*) to ten grams of both powdered samples. They were first sonicated in an ice bath for 10 min using a titanium probe sonicator set to 200 W and a 30% amplitude (Vibra Cell™ Sonics Materials, inc., Danbury, CT, USA), and then macerated under continuous stirring in the dark at RT for 2 h. Supernatants were recovered by filtration on Whatman paper filter n. 1. The extraction process was repeated twice. Collected supernatants were finally dry-evaporated by a rotary evaporator (Büchi R-205, Cornaredo, Italy) in the dark at 37 °C, and stored overnight in a vacuum glass desiccator with anhydrous sodium sulphate. The extraction yields were 17.15% and 18.60% for SsM and SsE, respectively. Dry extracts were then suspended and properly diluted in the same hydroalcoholic mixture reported above for phytochemical and biological analyses.

### 4.5. Phytochemical Screening

#### 4.5.1. Total Phenols

Total phenols were quantified according to Smeriglio et al. [60] by adding 50 µL of SsM and SsE hydroalcoholic extracts (0.625–5.0 mg/mL) to 450 µL deionised water and 500 µL Folin–Ciocalteu reagent. After 3 min incubation, 500 µL 10% sodium carbonate was added, incubating the samples in the dark at RT for 60 min and vortex mixing every 10 min. Absorbance was read at 785 nm (UV-1601, Shimadzu, Kyoto, Japan) against a blank consisting of the same hydroalcoholic mixture (ethanol:water, 80:20 *v*/*v*) used for resuspending the SsM and SsE extracts. Gallic acid was used as a reference compound (0.075–0.6 mg/mL), and the results were expressed as g gallic acid equivalents (GAE)/100 g dry extract (DE).

#### 4.5.2. Total Flavonoids

Total flavonoids were quantified according to Ingegneri et al. [61]. Briefly, 50 µL of SsM and SsE hydroalcoholic extracts (1.25–10 mg/mL) were added to 450 µL of deionised water. After this, 30 µL of 5% NaNO_2_ was added, and samples were incubated for 5 min at RT before adding 60 µL of 10% AlCl_3._ After 6 min, 200 µL of 1 M NaOH and 210 µL of deionised water were added. Samples were vortex mixed, and the absorbance was recorded at 510 nm using the same instrument and blank reported in Section 4.5.1. Rutin was used as the reference standard (0.125–1.0 mg/mL) and results were expressed as g rutin equivalents (RE)/100 g DE.

#### 4.5.3. Vanillin Index

This test is based on the ability of the vanillin aldehyde to react in an acid environment with the free carbons C6 and C8 of flavan-3-ols, leading to the formation of a red complex with a maximum absorbance at 500 nm [62]. Briefly, 2.0 mL of SsM and SsE hydroalcoholic extracts, diluted in 0.5 M H_2_SO_4_ to obtain a final absorbance between 0.2 and 0.4, were loaded onto a conditioned Sep-Pak C18 cartridge (Waters, Milan, Italy), washed with 2.0 mL of 5.0 mM H_2_SO_4_, and eluted with 5.0 mL of methanol. One millilitre of each eluate was added to 6.0 mL of 4% vanillin solution and incubated in a water bath at 30 °C for 10 min. After cooling, 3 mL of HCl was added. The absorbance was recorded after 15 min at 500 nm using the same instrument and blank reported in Section 4.5.1. Catechin was used as a reference compound (0.125–0.50 mg/mL). Results were expressed as g catechin equivalents (CE)/100 g DE.

#### 4.5.4. Proanthocyanidins

This method determines the proanthocyanidins content indirectly, by transforming them, by hot-hydrolyzation in an acid environment, in anthocyanidins [63]. Briefly, 40 mg of SsM and SsE hydroalcoholic extracts diluted in 0.05 M H_2_SO_4_ (2.0 mL) were loaded onto a conditioned Sep-Pak C18 cartridge (Waters, Milan, Italy). The proanthocyanidin-rich fraction obtained was eluted with methanol (3.0 mL) and collected in a 100 mL round bottom flask shielded from light and containing 9.5 mL of absolute ethanol. Thereafter, 12.5 mL of FeSO_4_·7H_2_O in concentrated HCl (300 mg/L) was added. Samples were refluxed for 50 min. After cooling, the absorbance was recorded at 550 nm by using the same instrument reported in Section 4.5.1. and by subtracting the basal anthocyanidins content, obtained by processing the samples in the same manner but without heating. The proanthocyanidin content was expressed as five times the amount of cyanidin formed by means of a cyanidin chloride (ε = 34,700) calibration curve. Results were expressed as g of cyanidin equivalents (CyE)/100 g DE.

### 4.6. Phytochemical Characterisation by LC-DAD-ESI-MS Analysis

The phytochemical characterisation of SsM and SsE hydroalcoholic extracts was carried out by LC-DAD-ESI-MS analysis according to Smeriglio et al. [64]. Chromatographic separation was carried out at RT using a reverse phase column (Luna Omega PS C18, 150 mm × 2.1 mm, 5 µm; Phenomenex, Torrance, CA, USA) and a mobile phase consisting of 0.1% HCOOH (Solvent A) and CH_3_OH (Solvent B) according to the following elution program: 0–3 min, 0% B; 3–9 min, 3% B; 9–24 min, 12% B; 24–30 min, 20% B; 30–33 min, 20% B; 33–43 min, 30% B; 43–63 min, 50% B; 63–66 min, 50% B; 66–76 min, 60% B; 76–81 min, 60% B; 81–86 min, 0% B, and equilibrated 4 min. The injection volume was 5 µL. The UV–Vis spectra were recorded ranging from 190 to 600 nm. Chromatograms were acquired at different wavelengths (260, 280, 292, 330, 370, and 520 nm) to identify all polyphenol classes. An ion trap (model 6320, Agilent Technologies, Santa Clara, CA, USA) coupled with an electrospray ionisation source (ESI) operating both in negative and positive ionisation mode was used by setting the parameters as follows: 3.5 kV capillary voltage, 40 psi nebuliser (N_2_) pressure, 350 °C drying gas temperature, 9 L/min drying gas flow, and 40 V skimmer voltage. The acquisition was carried out in full-scan mode (90–1000 m/z). Data were acquired by Agilent ChemStation software version B.01.03 and Agilent trap control software version 6.2.

### 4.7. Antioxidant and Anti-Inflammatory Activity

The antioxidant and anti-inflammatory activity of SsM and SsE hydroalcoholic extracts was evaluated by several in vitro colorimetric assays based on different mechanisms and reaction environments. The results, which represent the average of three independent experiments in triplicate (*n* = 3), were expressed as the inhibition (%) of the oxidative/inflammatory activity, calculating the IC_50_ with the respective C.L. at 95% by Litchfield and Wilcoxon’s test using PHARM/PCS software version 4 (MCS Consulting, Wynnewood, PA, USA). All concentration ranges reported below refer to the final concentrations of SsM and SsE hydroalcoholic extracts and reference compounds within the reaction mixture.

#### 4.7.1. TEAC Assay

The TEAC assay was carried out according to Ingegneri et al. [61]. The radical reagent was prepared by mixing 1.7 mM ABTS with 4.3 mM K_2_S_2_O_8_ and incubating for 12 h at RT in the dark. The radical solution was then diluted to obtain an average absorbance of 0.7 at 734 nm and used within 4 h. Ten microliters of SsM and SsE hydroalcoholic extracts (30–240 µg/mL and 60–480 µg/mL, respectively) were added to the reagent (200 µL) and incubated at RT for 6 min. The absorbance was recorded at 734 nm by using a UV–Vis reader plate (Multiskan GO; Thermo Scientific, Waltham, MA, USA) and the same blank reported in Section 4.5.1. Trolox was used as a reference compound (1.25–10.0 µg/mL). Results were expressed as reported in Section 4.7.

#### 4.7.2. FRAP Assay

The FRAP assay was carried out according to Ingegneri et al. [61]. Briefly, 10 µL of SsM and SsE hydroalcoholic extracts (30–240 µg/mL and 60–480 µg/mL, respectively) were added to 200 µL of fresh, pre-warmed (37 °C) working reagent consisting of 300 mM buffer acetate (pH 3.6), 10 mM TPTZ-40 mM HCl, and 20 mM FeCl_3_ and incubated for 4 min at RT in the dark. The absorbance was recorded at 593 nm using the same instrument and blank reported in Section 4.7.1. Trolox was used as a reference compound (1.25–10.0 µg/mL). Results were expressed as reported in Section 4.7.

#### 4.7.3. DPPH Assay

The DPPH assay was carried out according to Ingegneri et al. [61]. Briefly, 3.75 µL of SsM and SsE hydroalcoholic extracts (60–480 µg/mL) were added to 150 µL fresh DPPH methanol solution (70 mg/L), mixed and incubated in the dark for 20 min. The absorbance was recorded at 517 nm using the same instrument and blank reported in Section 4.7.1. Trolox was used as the reference standard (2.5–20.0 µg/mL). Results were expressed as reported in Section 4.7.

#### 4.7.4. ORAC

The ORAC assay was carried out according to Danna et al. [65]. Briefly, 20 µL of SsM and SsE hydroalcoholic extracts (0.38–3.0 µg/mL and 0.75–6.0 µg/mL, respectively) diluted in 75 mM PBS at pH 7.4 was added to 120 µL of fresh 117 nM fluorescein and incubated 15 min at 37 °C. Sixty microliters of 40 mM AAPH radical were added to start the reaction, which was monitored every 30 s for 90 min (λ_ex_ 485; λ_em_ 520) by a fluorescence reader plate (FLUOstar Omega, BMG LABTECH, Ortenberg, Germany) against the same blank reported in Section 4.5.1. Trolox was used as the reference standard (0.25–2.0 µg/mL). Results were expressed as reported in Section 4.7.

#### 4.7.5. ICA Assay

The iron-chelating activity was evaluated according to Bazicalupo et al. [66]. Briefly, 50 µL of 2.0 mM FeCl_2_ • 4 H_2_O was added to 100 µL of SsM and SsE hydroalcoholic extracts (20–160 µg/mL) and incubated at RT for 5 min. After that, 100 µL of 5 mM ferrozine was added to the reaction mixture, and the sample solution was diluted to 3 mL with deionised water, mixed, and incubated for 10 min at RT. The absorbance was read at 562 nm using the same instrument and blank reported in Section 4.5.1. EDTA was used as the reference standard (1.5–12.0 µg/mL). Results were expressed as reported in Section 4.7.

#### 4.7.6. BCB Assay

The BCB assay was carried out according to Smeriglio et al. [67]. Briefly, 0.320 mL of SsM and SsE hydroalcoholic extracts (50–400 µg/mL) and reference standard (BHT, 0.06–0.5 μg/mL) were added to 8 mL of a β-carotene emulsion consisting of β-carotene chloroform solution (1 mg/mL), 40 μL of linoleic acid, and 400 μL of Tween-40. An emulsion without β-carotene was used as a negative control, whereas a β-carotene emulsion with a sample solvent (ethanol:water, 80:20 *v*/*v*) was used as a blank. The absorbance was monitored every 20 min for 120 min at 50 °C by recording the absorbance decay at 470 nm using the same instrument reported in Section 4.5.1.

#### 4.7.7. Protease Assay

The protease inhibitory activity was evaluated according to Cornara et al. [11]. Briefly, 200 µL of SsM and SsE hydroalcoholic extracts (12.50–100 µg/mL and 25.0–200 µg/mL, respectively) were added to a reaction mixture consisting of 12 µL trypsin (10 µg/mL) and 188 µL Tris-HCl buffer (25 mM, pH 7.5). Two-hundred microliters of 0.8% casein were added and the reaction mixture and incubated for 20 min at 37 °C in a water bath. The reaction was stopped by adding 400 µL of perchloric acid. The cloudy suspension was centrifuged at 3500× *g* for 10 min, and the absorbance of the supernatant was recorded at 280 nm using the same instrument and blank reported in Section 4.5.1. Diclofenac sodium was used as the reference standard (5.0–40.0 µg/mL). Results were expressed as reported in Section 4.7.1.

#### 4.7.8. ADA Assay

The ability of the two hydroalcoholic extracts to inhibit heat-induced albumin denaturation was evaluated according to Cornara et al. [11]. Briefly, 100 µL of 0.4% fatty-acid-free bovine serum albumin solution and 20 µL of PBS (pH 5.3) were seeded in a 96-well plate. Then, 80 µL of SsM and SsE hydroalcoholic extracts (25.0–200 µg/mL, respectively, and 50.0–400 µg/mL) were added, and the absorbance was immediately recorded at 595 nm. Subsequently, samples were incubated for 30 min at 70 °C, and, in the end, the absorbance was recorded again using the same instrument and blank reported in Section 4.7.1. Diclofenac sodium was used as a reference compound (3.0–24.0 µg/mL). Results were expressed as reported in Section 4.7.

### 4.8. Statistical Analysis

Nine independent evaluations in triplicate (*n* = 3) for micromorphological analysis and three independent analyses/experiments in triplicate (*n* = 3) for phytochemical and biological studies were carried out. The statistical significance was evaluated by a one-way analysis of variance (ANOVA) followed by a Student–Newman–Keuls and Tukey’s test using SigmaPlot 12.0 software (Systat Software Inc., San Jose, CA, USA). *p* < 0.05 was considered statistically significant.

## 5. Conclusions

This is the first study that investigates the micromorphological and phytochemical features, as well as the biological activity, of the rare Sicilian endemic species *Saponaria sicula* Raf. Moreover, this is the first study that, using a multidisciplinary approach, investigates the possible influence of different pedo-climatic conditions on the characteristics of this plant species.

Micromorphological investigations revealed that the leaves from SsM had a higher amount of calcium oxalate druses in the mesophyll, probably due to the soil which is richer in CaCO_3_, Ca, and Mg, as well as in organic matter with respect to the Etna soil. Furthermore, the leaf mesophyll of SsM showed more intense blue-greenish staining with TBO, indicating a higher content of polyphenols. These data were confirmed by phytochemical analyses carried out on leaf hydroalcoholic extracts which showed a higher content of total phenols, flavonoids, and flavan-3-ols in SsM, with a preponderance of monomeric compounds. LC-DAD-ESI-MS analysis, while showing a similar qualitative phytochemical profile as expected from two extracts of the same plant species, highlighted a statistically significant difference in terms of the secondary metabolite expression between the two investigated extracts. Finally, the higher polyphenol content of SsM also correlated with the results of the biological assays, identifying the SsM extract as the strongest plant complex.

In conclusion, the present study experimentally demonstrates that not only climatic differences but also soil characteristics affect the micromorphological, phytochemical, and biological features of this plant species.

## Figures and Tables

**Table 1:** Soil features of the two collection sites.

Soil Features	Madonie	Etna
Sand %	79.9	94.6
Silt %	19.0	1.3
Clay %	1.1	4.1
pH	8.3	7.5
Total N (g/kg)	1.03	0.75
CaCO_3_ g/kg	916.8	4.5
O.M. ^a^ (g/100)	2.4	0.9
C/N	13.6	7.1
Cond. ^b^ (1:5 mS/cm)	0.07	0.05
C.E.C ^c^ (meq/100 g)	6.9	4.9
Ca ^d^ (mg/kg)	860.0	50.0
Mg ^d^ (mg/kg)	135.6	8.4
K ^d^ (mg/kg)	54.7	74.3

^a^ O.M.: organic matter; ^b^ Cond.: electrical conductivity; ^c^ C.E.C.: cation exchange capacity; and ^d^ exchangeable.

**Table 2:** Intra- and inter-specific differences in the stomatal index (%) and density (n. of stomata per mm^2^) in *S. sicula* leaves collected on Madonie and Etna (SsM and SsE, respectively). Results are expressed as the mean ± standard deviation (*n* = 9).

	SsM		SsE	
	Abaxial	Adaxial	Abaxial	Adaxial
Stomatal Index (%)	25 ± 2	26 ± 3	24 ± 2	29 ± 5
Stomatal Density (stomata/mm^2^)	102 ± 9 ^a,c^	140 ± 22 ^d^	171 ± 43 ^b^	208 ± 59

^a^ *p* < 0.001 vs. SsM adaxial; SsE: ^b^ *p* > 0.01 vs. SsM adaxial; ^c^ *p* < 0.001 vs. SsE abaxial; and ^d^ *p* < 0.01 vs. SsE adaxial.

**Table 3:** Phytochemical screening of the hydroalcoholic extracts of *S. sicula* collected on Madonie and Etna (SsM and SsE, respectively). Results are the mean ± standard deviation of three independent experiments in triplicate (*n* = 3).

Phytochemical Assays	SsM	SsE
Total phenols (g GAE ^a^/100 g DE ^b^)	8.56 ± 0.57 ^g^	6.54 ± 0.16
Flavonoids (g RE ^c^/100 g DE)	6.09 ± 0.17 ^g^	5.31 ± 0.32
Vanillin index (g CE ^d^/100 g DE)	0.60 ± 0.02 ^g^	0.28 ± 0.08
Proanthocyanidins (g CyE ^e^/100 g DE)	0.04 ± 0.06	0.05 ± 0.08
Polymerisation index ^f^	15.00	5.60

^a^ GAE, Gallic acid equivalents; ^b^ DE, Dry extract; ^c^ RE, Rutin equivalents; ^d^ CE, Catechin equivalents, ^e^ CyE, Cyanidin equivalents; ^f^ Polymerisation index = vanillin index/proanthocyanidins; and ^g^ *p* < 0.05 vs. SsE.

**Table 4:** Tentative identification of the secondary metabolite profile of *S. sicula* leaves collected on Madonie and Etna (SsM and SsE, respectively) by LC-DAD-ESI-MS analysis. Results were expressed as the mean area (%) based on LC-ESI-MS data ± standard deviation of three independent analyses in triplicate (*n* = 3) with respect to the total area of detected compounds.

Compound	RT ^a^	λ_max_ (nm)	[M-H]^−^	[M-H]^+^	SsM	SsE
					Area%	
*p*-Coumaroyl glucose ^d^	7.4	322	-	327	1.59 ± 0.06 ^b^	3.30 ± 0.15
Acetylglycitin ^d^	11.2	264, 324	-	489	0.57 ± 0.02 ^b^	0.82 ± 0.04
Eriodictyol-*O*-glucoside ^c^	13.3	289, 328	-	451	0.58 ± 0.02	0.55 ± 0.03
Dihydroxy-dimethoxy-methylenedioxyflavone-O-glucuronide	15.1	247, 274, 315, 342	533	-	0.07 ± 0.00 ^b^	0.61 ± 0.02
Feruloylquinic acid ^d^	17.3	290, 322	-	369	-	0.54 ± 0.03
Tricosylresorcinol	18.9	273	-	433	2.64 ± 0.08 ^b^	0.56 ± 0.03
Delphinidin-*O*-(acetyl-glycoside) ^d^	19.6	245, 529	506	-	0.06 ± 0.00	-
Valoneic acid dilactone	20.2	256, 305, 347, 362	468	-	0.93 ± 0.04 ^b^	0.66 ± 0.02
Ellagic acid arabinoside ^d^	20.4	254, 360	433	-	0.10 ± 0.01	0.08 ± 0.00
Apigenin-*O*-glucuronide ^d^	21.3	267, 336	-	447	-	0.99 ± 0.03
Luteolin-*O*-malonyl-glucoside ^d^	21.4	255, 267, 348	533	-	0.10 ± 0.01	0.08 ± 0.00
(-)-Epigallocatechin ^c^	21.5	240, 274	-	307	1.47 ± 0.03	-
Geranylnaringenin ^d^	22.6	289, 326	-	409	1.02 ± 0.02 ^b^	0.66 ± 0.01
Methylepigallocatechin-*O*-gallate ^d^	22.7	232, 274	-	473	0.69 ± 0.03	0.70 ± 0.02
Pallidol	24.1	203, 230, 324	453	-	0.06 ± 0.00	-
(+)-Catechin-*O*-glucose ^d^	25.0	238, 269	-	453	1.63 ± 0.05 ^b^	1.10 ± 0.08
Kaempferol-*O*-(acetyl-galactoside)-*O*-rhamnoside ^d^	25.7	245, 265, 315, 350	635	-	0.06 ± 0.00	0.06 ± 0.00
Apigenin-*O*-diglucuronide ^d^	25.8	267, 336	-	623	1.02 ± 0.02	0.96 ± 0.03
Methyl-(-)-epicatechin-*O*-glucuronide ^d^	27.1	240, 274	-	481	1.89 ± 0.03 ^b^	1.32 ± 0.04
Ligstroside	29.0	235, 275	-	525	2.28 ± 0.08 ^b^	1.52 ± 0.05
Phloretin-*O*-xylosyl-glucoside ^d^	30.2	242, 289	-	541	2.53 ± 0.12	-
Apigenin-*O*-(malonyl-apiosyl-glucoside) ^d^	30.5	268, 333	-	651	-	2.80 ± 0.10
Hydroxyphloretin-*O*-xylosyl-glucoside ^d^	30.9	242, 289	-	585	2.44 ± 0.08 ^b^	2.80 ± 0.12
Quercetin-*O*-alpha-L-rhamnopyranosyl(1-2)-beta-D-glucopyranoside-*O*-alpha-L-rhamnopyranoside ^d^	31.0	258, 272, 303, 365	755	-	0.11 ± 0.00	0.12 ± 0.01
Hydroxyphloretin-*O*-glucoside ^d^	31.4	242, 289	-	585	3.58 ± 0.15 ^b^	3.07 ± 0.18
Kaempferol-*O*-glucosyl-rhamnosyl-glicoside ^d^	31.6	253, 265, 325, 364	755	-	0.11 ± 0.00	0.10 ± 0.00
Patuletin-gentiobioside	32.2	261, 270, 355	-	657	1.75 ± 0.11 ^b^	1.34 ± 0.08
Prodelphinidin dimer B3	32.6	228, 276, 320	609	-	0.30 ± 0.02 ^b^	0.42 ± 0.02
Isorhamnetin-*O*-glicoside ^c^	32.7	255, 268, 303, 357	-	479	2.03 ± 0.05	2.07 ± 0.08
Saponarin	33.6	271, 336	593	-	0.11 ± 0.01	0.09 ± 0.00
Kaempferol-*O*-xylosyl-glucoside ^d^	33.7	253, 266, 323, 364	-	581	1.90 ± 0.08 ^b^	3.47 ± 0.14
Quercetin-*O*-diglucoside ^d^	33.9	256, 362	625	-	-	0.09 ± 0.00
Isorhamnetin-*O*-glucoside-*O*-rhamnoside ^d^	34.4	254, 265, 305, 356	-	625	3.56 ± 0.15 ^b^	0.47 ± 0.02
Isorhamnetin-*O*-glicoside ^c^	34.8	255, 268, 303, 357	-	479	-	1.96 ± 0.07
Propelargonidin dimer	36.0	245, 280	-	563	10.49 ± 0.35	10.19 ± 0.22
Hydroxyphloretin-*O*-xylosyl-glucoside ^d^	36.8	242, 289	-	585	0.06 ± 0.00	-
Sinapoyl-feruloylgentiobiose	38.6	282	723	-	6.54 ± 0.25 ^b^	7.43 ± 0.36
Diosmin ^c^	40.3	260, 350	-	609	2.24 ± 0.11	2.16 ±0.08
Luteolin-*O*-diglucuronide ^d^	40.4	245, 253, 267, 292, 348	637	-	0.01 ± 0.00	0.09 ± 0.00
Saponarioside K	41.2	-	988	-	0.12 ± 0.01 ^b^	0.17 ± 0.01
Sinapoyl-diferuloylgentiobiose	42.7	282	-	901	1.40 ± 0.10	-
Kaempferol-*O*-feruloyl-sophoroside ^d^	43.4	245, 265, 317, 350	-	787	3.51 ± 0.15	3.83 ± 0.22
Saponarioside J	44.4	-	-	1104	2.41 ± 0.08 ^b^	1.67 ± 0.05
Petunidin ^c^	45.8	279, 524	-	318	5.52 ± 0.22 ^b^	3.91 ± 0.17
Peonidin-*p*-hydroxybenzoylsophoroside-glucoside ^d^	46.4	275, 500	906	-	0.09 ± 0.00 ^b^	2.94 ± 0.12
Pelargonidin-diglucoside- (malonyl)-glucoside ^d^	46.8	267, 500	842	-	0.10 ± 0.00 ^b^	0.77 ± 0.02
Phloretin ^c^	47.1	242, 289	-	275	3.52 ± 0.21	3.77 ± 0.18
Saponarioside H	47.4	-	779	-	0.09 ± 0.00 ^b^	2.47 ± 0.12
Dihydromyricetin-*O*-rhamnoside ^d^	48.1	254, 274, 303, 374	-	467	2.08 ± 0.12 ^b^	1.60 ± 0.08
Saponarioside I	48.2	-	1282	-	-	0.09 ± 0.00
Disinapoyl-feruloylgentiobiose	48.8	238, 289, 320	929	-	0.09 ± 0.00	0.08 ± 0.00
Quercetin-di-glucoside-(caffeoylglucoside) ^d^	49.7	257, 271, 301, 362	949	-	0.09 ± 0.00 ^b^	1.89 ± 0.08
Caffeoyl glucose ^d^	50.6	290, 305, 328	342	-	5.60 ± 0.23 ^b^	4.33 ± 0.16
Dihydrocaffeic acid-*O*-glucuronide ^d^	51.4	240, 300, 324	357	-	-	0.20 ± 0.01
Tigloylgomicin H	52.8	230, 280	-	501	-	1.53 ± 0.06
Coumaroylquinic acid ^d^	53.8	280, 308, 320	337	-	0.13 ± 0.01	0.13 ± 0.01
Resveratrol-*O*-glucoside ^d^	55.0	289	389	-	0.12 ± 0.01	0.10 ± 0.01
Methylepicatechin ^d^	55.9	238, 274	-	305	2.55 ± 0.13 ^b^	1.88 ± 0.06
Phloridzin ^c^	58.2	230, 285	-	437	-	1.62 ± 0.04
Quercetin-sulfate ^d^	61.6	255, 270, 303, 370	-	383	3.35 ± 0.21 ^b^	2.28 ± 0.16
Hydroxymatairesinol	63.2	230, 280	-	375	4.81 ± 0.24 ^b^	3.38 ± 0.17
Baicalin ^c^	64.2	244, 278, 315	-	447	4.45 ± 0.28 ^b^	3.44 ± 0.15
Lariciresinol-sesquilignan	72.9	230, 280	-	557	5.47 ± 0.28 ^b^	4.72 ± 0.14

^a^ RT, Retention time; ^b^ *p* < 0.05 vs. SsE; ^c^ comparison of the UV–Vis and mass spectra with commercially available reference standards; and ^d^ comparison of the UV–Vis spectra with structurally similar reference standards.

**Table 5:** Antioxidant and anti-inflammatory properties of the leaf hydroalcoholic extracts of *S. sicula* coming from Madonie and Etna (SsM and SsE, respectively) by several in vitro colorimetric assays based on different environments and reaction mechanisms. Data, expressed as half-maximal inhibitory concentrations (IC_50_, μg/mL) with 95% confidence limits (C.L.) are the mean of three independent experiments in triplicate (*n* = 3).

Assay	SsM	SsE	RS ^c^
Trolox equivalent antioxidant capacity (TEAC)	99.75 ^a,b^ (77.29–128.73)	191.07 ^b^ (143.65–254.15)	4.03 (1.72–9.45)
Ferric reducing antioxidant power (FRAP)	165.72 ^a,b^ (137.31–199.99)	254.90 ^b^ (207.04–313.84)	3.69 (1.61–8.45)
2,2-Diphenyl-1-picrylhydrazyl (DPPH)	477.30 ^b^ (373.40–610.12)	478.92 ^b^ (372.95–614.99)	10.38 (8.82–12.22)
Oxygen radical absorbance capacity (ORAC)	2.75 ^b^ (2.22–3.42)	4.04 ^b^ (3.36–4.85)	0.67 (0.32–0.88)
Iron-chelating activity (ICA)	129.77 ^b^ (104.57–161.04)	144.24 ^b^ (112.47–184.99)	5.51 (2.46–12.32)
β-arotene bleaching (BCB)	180.96 ^a,b^ (147.51–221.98)	282.57 ^b^ (229.77–363.32)	0.32 (0.15–0.55)
Albumin denaturation assay (ADA)	195.60 ^a,b^ (154.03–248.40)	390.22 ^b^ (299.15–509.03)	11.16 (9.25–13.46)
Protease inhibitory activity	43.48 ^b^ (34.99–54.04)	61.53 ^b^ (51.07–74.14)	18.97 (14.33–25.11)

^a^ *p* < 0.05 vs. SsE; ^b^ *p* < 0.05 vs. RS; ^c^ RS, reference standard which is trolox for the DPPH, TEAC, FRAP, and ORAC assays, butylated hydroxytoluene (BHT) for BCB, ethylenediaminetetraacetic acid (EDTA) for iron-chelating activity (ICA), and diclofenac sodium for the ADA and protease assays.

**Table 6:** Collection site details of the two populations of *S. sicula*.

Population	Collection Site	Coordinates	Altitude	Exposure	Substrate	SAF Code
Madonie	Quacella	37°50′48.61′′ N 14°0′55.02′′ E	1350	W	carbonate	100081
Etna Mt	Piano Provenzana	37°47′56.53′′ N 15°02′44.88′′ E	1800	S	volcanic	100082

## Data Availability

Data are contained within the article.
